# Early prediction of urinary tract infection in neonates with hyperbilirubinemia

**DOI:** 10.12861/jrip.2015.18

**Published:** 2015-09-01

**Authors:** Azar Nickavar, Nastaran Khosravi, Mahdiye Doaei

**Affiliations:** ^1^Department of Pediatric Nephrology, Iran University Medical Sciences, Tehran, Iran; ^2^Department of Neonatology, Iran University Medical Sciences, Tehran, Iran; ^3^Departmet of Community Medicine, Iran University Medical Sciences, Tehran, Iran

**Keywords:** Urinary tract infection, Neonates, Jaundice

## Abstract

**Introduction:** Hyperbilirubinemia is a common manifestation of infectious disorders during the neonatal period. Urinary tract infection (UTI) is one of the serious bacterial infections with hyperbilirubinemia among newborn infants.

**Objectives:** The aim of this study was to identify the early predictive risk factors of UTI in neonates with hyperbilirubinemia, to prevent its long-term complications.

**Patients and Methods:** A total of 95 neonatal hyperbilirubinemia were evaluated in 2 groups with (n = 40) and without UTI (n = 55).

**Results:** Mean age at diagnosis of UTI was 16.37 ± 8.86 days. Hyperbilirubinemia was detected in 70% of patients during the first week of life. There was a significant difference regarding the age at admission, duration of hyperbilirubinemia, serum bilirubin and creatinine, white blood cells (WBC) , and also Hgb levels between the 2 groups in univariate analysis. However, prolonged jaundice (OR = 10.3, *P* = 0.001) and serum bilirubin concentration (OR = 5.15, *P* = 0.001) were statistically associated with a positive urine culture in multivariate analysis.

**Conclusion:** Screening of UTI is recommended in neonates with prolonged unexplained jaundice, leukocytosis, and increased serum creatinine.

**Implication for health policy/practice/research/medical education:**

We recommend screening of urinary tract infection (UTI) in jaundiced neonates with prolonged unexplained hyperbilirubinemia, leukocytosis, and increased serum creatinine.

## Introduction

Urinary tract infection (UTI) is a common clinical problem in children of all age groups, consists 1/3 of bacterial infections in newborn infants. Appropriate diagnosis and treatment prevent complications such as urosepsis, hypertension, proteinuria and end stage renal disease (1,2).

Immaturity of local immunity including low uroepithelial bactericidal activity, low secretory IgA level, decreased renal acidification and severe periurethral colonization are the major risk factors for increased susceptibility to UTI in the neonatal period (3).

Association between UTI and hyperbilirubinemia has been explained by Gorter and Lignac in 1928 (4). Jaundice may be the first or major manifestation of UTI in the neonatal period (5,6). UTI may occur in 3%-21% of term neonates with unexplained indirect hyperbilirubinemia during the first 2 weeks of life (7). Hemolysis, hepatocellular damage with impaired bilirubin conjugation and excretion, fever and malnutrition are the pathophysiologic mechanisms of hyperbilirubinemia in UTI (4). Previous studies suggested the investigation of UTI in neonates with late onset jaundice, prolonged jaundice and asymptomatic indirect hyperbilirubinemia during the first 2 weeks of life (6-8).

## Objectives

This study was performed to identify the predictive risk factors of UTI in neonates with hyperbilirubinemia to identify those requiring early urine culture and treatment strategies.

## Patients and Methods

### Subjects

This study was conducted on all neonates with UTI (3-28 days) admitted (n = 40) in neonatal intensive care unit (NICU) of Aliasghar children’s hospital between 2005 and 2014. All patients admitted during the study period were out born. Data were collected from NICU and nephrology clinic medical records. Fifty-five healthy neonates with unexplained indirect hyperbilirubinemia and negative urine culture titled as the control group.

Demographic characteristics including prenatal history [maternal disorders, premature rupture of membrane, preeclampsia, oligohydramniosis, type of delivery], neonatal history [gestational age, gender, birth weight, age at admission], feeding pattern [breast feeding; more than 50% of intake], clinical manifestations of UTI [fever, vomiting, diarrhea, lethargy, irritability, poor feeding, failure to thrive] and laboratory exams [CBC, ESR, CRP, total and direct bilirubin level, serum creatinine, blood culture, urinalysis and urine culture] were obtained.

Patients with a history of previous antibiotic treatment, hemolytic disorders, large cephalohematoma or ecchymosis, gastrointestinal obstruction, multiple congenital anomalies, UTI with contaminated organisms, and other pathologic causes of indirect hyperbilirubinemia were excluded.

Hyperbilirubinemia referred to serum bilirubin level more than 10 mg/dl in 2 groups as early onset during the first week and late onset after the eighth day of life. Prolonged hyperbilirubinemia was considered as jaundice lasting for more than 14 days in full term infants. Serum creatinine was evaluated based on gestational and postnatal age.

UTI was defined as positive urine culture (any growth in suprapubic aspiration, >10^4^ by urethral catheterization or >10^5^ in bag collection of a single organism) associated with pyuria and bacteriuria. Pyuria referred to >5 white blood cells (WBC)/hpf of centrifuged urine and bacteriuria to at least 1 bacteria/hpf in an unspun urine sample. Patients were treated with ampicillin and aminoglycosides as the empiric treatment, adjusted according to the antibiotic susceptibility tests.

Ultrasonography using 7.5 MHz probe was performed in the acute phase of UTI in all patients. Hydronephrosis was defined as pelvic AP diameter >5 mm. Vesicoureteral reflux diagnosed by conventional or radionuclide cystography in clinically stable neonates >1500 g after treatment of UTI.

### Ethical issues

The research followed the tenets of the Declaration of Helsinki and approved by the ethical committee of Iran University of medical sciences. Informed consent was obtained, and parents were free to leave the study at any time.

### Statistical analysis

Data were analyzed by SPSS version 21 using independent *t* test for comparing quantitative variables and chi-square test for comparing qualitative variables. Receiver operating characteristic (ROC) curve and chi-square were used for calculating and displaying sensitivity and specificity.

## Results

### Common results

Mean age at diagnosis of UTI was 16.37 ± 8.86 days. Males outnumbered females (1.8/1). Hyperbilirubinemia was detected in 70% of them during the first week of life. Majority of neonates with UTI had irritability 25%, followed by poor feeding 17%, lethargy 15%, vomiting 15.2%, fever 12.5%, tachypnea 10.3%, and diarrhea 5%. Leukocytosis >13 000/mm^3^ and positive CRP were the most frequent laboratory findings, respectively.

None of the patients had concomitant positive blood culture with the same organism. *Escherichia coli* (*E. coli*) 36.8%, klebsiella 10.5%, enterobacter 5.3% and enterococcus 3.6% were the most common isolated organisms, respectively. *E. coli* was more common in both terms and premature newborns in our study.

Ultrasonography showed urinary tract abnormality in 37.5% of patients. Majority of them were males with *E. coli* as the major isolated organism. Bilateral hydronephrosis was the most common renal abnormality in 12.8% of patients. Cystography was performed in 42% of patients, which showed vesicoureteral reflux in 64.7%, and mostly of low grade. Clinical and demographic characteristics of neonates with UTI are presented in Table 1.

**Table 1:** Clinical and demographic characteristics of neonates with urinary tract infection

**Variables**	**No.**	**%**	**Variables**	**No.**	**%**
GA (wk)			Urine SG		
Term ≥37	35	87.5	NL	23	69.6
Premature <37	5	12.5	Abn	10	30.4
BW (g)			Urine PH		
<2500	6	15	NL	36	90
>2500	34	85	Abn	4	10
WBC (mm^3^)			Hematuria		
<13000	29	72.5	Positive	15	37.5
>13000	11	27.5	Negative	25	62.5
Hgb (g/d)l			CRP		
NL	23	57.5	Positive	7	17.5
Abn	17	42.5	Negative	33	82.5
Plt (µl)			Prolonged jaundice		
<150 000	4	10	Positive	24	60
>150 000	36	90	Negative	16	40
BUN (mg/dl)			Bacteriuria		
<40	39	97.5	Positive	18	45
>40	1	2.5	Negative	22	55
Cr (mg/dl)			Pyuria		
<0.4	23	57.5	Positive	34	85
>0.4	17	42.5	Negative	6	15

Abbreviations‏: GA, gestational age; BW, birth weight; NL, normal; Abn, abnormal; Plt, platelet; Cr, creatinine

### Specific results

There was no significant difference in terms of gender, complications of pregnancy, exposure to breast milk, type of delivery, blood group, platelet count, urine specific gravity, urine PH, blood urea nitrogen and direct bilirubin level between the 2 groups of patients. Age at admission, duration of hyperbilirubinemia, total serum bilirubin, serum creatinine, WBC and Hgb were significant predictive variables in univariate analysis. However, prolonged jaundice (OR = 10.3, *P *= 0.001) and total serum bilirubin (OR = 5.15, *P* = 0.001) were independently associated with UTI in multivariable analysis (Tables 2 and 3).

**Table 2:** Quantitative variables in patients with and without urinary tract infection

**Variables**	** UTI**			**Isolated hyper BR**																															
	** Mean**	**SD**	**Mean**	**SD**	***P value***
Age at admission (day)	16.37	8.86	6.98	3.86	0.001
Age at jaundice (day)	3.82	3.06	3.56	2.17	0.659
Birth weight (g)	3176.7	647.92	3054.09	558.44	0.326
BR level (mg/dl)	14.54	3.01	17.96	2.51	0.001
Direct BR (mg/dl)	0.46	0.14	0.44	0.15	0.569
WBC (mm^3^)	10970	3367.73	9292	3101.01	0.014
Hgb (g/dl)	13.05	2.5	14.57	1.67	0.002
Platelet (µl)	333215	149213.21	298580	130820.22	0.232
BUN (mg/dl)	8	5.00	9.65	6.69	0.218
Cr (mg/dl)	0.55	0.26	0.43	0.16	0.014
Urine PH	5.83	0.86	6.09	0.59	0.123
Urine SG	1010.054	7.79	1010.745	6.9	0.529

Abbreviations: BR, bilirubin; Cr, creatinine; SG, specific gravity; UTI, urinary tract infection.

**Table 3:** Qualitative variables in patients with and without urinary tract infection

**Variables**	**UTI**		**Isolated hyper BR**		***P *** **value**	**Chi** ^2^
	**No.**	**%**	**No.**	**%**		
Gender					0.697	0.152
Male	26	65	35	63.6		
Female	14	35	20	36.4		
Feeding pattern					0.097	7.85
BF	31	88.6	51	92.7		
FF	2	5.7	1	1.8		
Mixed	2	5.7	3	5.5		
Delivery type					0.16	1.97
NVD	13	32.5	7	12.7		
C/S	27	67.5	48	87.3		
Mother disease					1.000	1.192
PROM	1	2.6	0	0		
Negative	30	78.9	47	85.5		
OHA	1	2.6	0	0		
Preeclampsia	2	5.3	0	0		
GDM	2	5.3	4	7.3		
Hyopthyroidism	0	0	2	3.6		
Hypertension	1	2.6	2	3.6		
HN	1	2.6	0	0		
Blood group					0.069	15.89
A	4	14.8	23	41.8		
B	11	40.7	13	23.6		
AB	3	11.1	1	1.8		
O	9	33.3	18	32.7		

Abbreviations: BF, breast feeding; FF, formula feeding; NVD, normal vaginal delivery; C/S, cesarian section; PROM, premature rupture of membrane; OHA, oligohydramniosis; GDM, gestational diabetes mellitus; HN, hydronephrosis.

WBC and serum creatinine had the most sensitivity (63.64% vs 60.87%) and age of admission the greatest specificity in diagnosis of UTI (92.31%). Age at admission and serum creatinine had the highest positive predictive value (96.43% vs 68.29%) and positive likelihood ratio (6.38 vs 1.45) in UTI diagnosis.

## Discussion

This study was performed to identify the predictive risk factors of UTI in neonates admitted with hyperbilirubinemia. Screening for UTI in jaundiced neonates have been controversial in the previous studies. The majority of neonates with UTI had prolonged hyperbilirubinemia with older age at admission compared to those without UTI in our study. In addition, total bilirubin level was significantly lower in neonates with UTI than non-UTI group. Similarly, Abourazzak et al (9), showed lower bilirubin level in neonates with UTI with no conjugated hyperbilirubinemia.

UTI rarely occur during the first day of life (10), and usually presents after 72 hours (11). 7.5% of our patients admitted during the first 24 hours of life. Mean age at diagnosis of UTI was 3.82 ±3.06 days and jaundice occurred in 70% of patients during the first week of life.

Similar to our study, male predominance have been reported in 82% of patients less than 8 weeks in the previous studies (8,12), for increased incidence of structural abnormalities, phimosis, periurethral colonization and increased sensitivity to bacterial infections (13). However, the incidence of UTI has been relatively the same in both genders in Hsieh et al study (14).

UTI occurred more commonly (10/1.45) in low birth weight infants compared to term neonates in Bilgen et al study (5). However, birth weight and gestational age had no significant correlation to UTI in our study.

Clinical manifestations of UTI are extremely variable in newborns. The majority of neonates are asymptomatic or have mild and non-specific symptoms such as fever, poor feeding, diarrhea, vomiting, lethargy, irritability and respiratory distress (15). However, symptomatic bacteriuria occurred in 1.9% and asymptomatic bacteriuria in 0.5% of neonates with UTI in Maherzi et al study (16). The majority of our patients had nonspecific symptoms such as irritability, poor feeding, and lethargy.

Increased serum creatinine might be a valuable marker for predicting renal parenchymal involvement in acute pyelonephritis. Our investigation showed that leukocytosis more than 13 000/mm^3^ and increased serum creatinine were significantly associated with UTI and consider the sensitive markers for predicting UTI.

Abnormal urinalysis have been reported in 50% of asymptomatic neonates with hyperbilirubinemia and UTI. Excluding urine culture in neonates with normal urinalysis, will miss more than 50% of infections. Eighty-five percent of our patients had pyuria with significant amount of WBC in 65% of them. However, urine PH and urine specific gravity had no significant differences between the 2 groups.

## Conclusion

The authors recommend routine urine culture in jaundiced neonates with leukocytosis, decreased Hgb level, increased serum creatinine and prolonged hyperbilirubinemia.

## Limitations of the study

There are some limitations in our study. It had a retrospective design with small sample size. But, we tried to collect all of the jaundiced neonates with UTI during the period of study. Although supra pubic aspiration has been considered the standard test for diagnosis of UTI, it was not performed in all patients for parent’s non-cooperation or failure of urine aspiration. However, we combined urinalysis associated with urine culture to enhance the reliability of our findings.

## Authors’ contribution

AN; data collection and article preparation. NK; data collection. MD; statistical analysis.

## Conflicts of interest

The authors declared no competitive interests.

## Ethical considerations

Ethical issues (including plagiarism, data fabrication, double publication) have been completely observed by the authors.

## Funding/Support

None.
